# Early Stages of Ex Vivo Collagen Glycation Disrupt the Cellular Interaction and Its Remodeling by Mesenchymal Stem Cells—Morphological and Biochemical Evidence

**DOI:** 10.3390/ijms25115795

**Published:** 2024-05-26

**Authors:** Regina Komsa-Penkova, Borislav Dimitrov, Svetla Todinova, Violina Ivanova, Svetoslava Stoycheva, Peter Temnishki, Galya Georgieva, Pencho Tonchev, Mario Iliev, George Altankov

**Affiliations:** 1 Department of Biochemistry, Medical University Pleven, 5800 Pleven, Bulgaria; 2 Institute of Biophysics and Biomedical Engineering, Bulgarian Academy of Sciences, 1113 Sofia, Bulgaria; 3 Department of Surgery, Medical University Pleven, 5800 Pleven, Bulgaria; 4 Faculty of Physics, Sofia University “St. Kliment Ohridski”, 1504 Sofia, Bulgaria; 5 Research Institute, Medical University Pleven, 5800 Pleven, Bulgaria

**Keywords:** collagen remodeling, ex vivo glycation, mesenchymal stem cell interaction

## Abstract

Mesenchymal stem cells (MSCs), pivotal for tissue repair, utilize collagen to restore structural integrity in damaged tissue, preserving its organization through concomitant remodeling. The non-enzymatic glycation of collagen potentially compromises MSC communication, particularly upon advancing the process, underlying various pathologies such as late-stage diabetic complications and aging. However, an understanding of the impact of early-stage collagen glycation on MSC interaction is lacking. This study examines the fate of in vitro glycated rat tail collagen (RTC) upon exposure to glucose for 1 or 5 days in contact with MSCs. Utilizing human adipose tissue-derived MSCs (ADMSCs), we demonstrate their significantly altered interaction with glycated collagen, characterized morphologically by reduced cell spreading, diminished focal adhesions formation, and attenuated development of the actin cytoskeleton. The morphological findings were confirmed by ImageJ 1.54g morphometric analysis with the most significant drop in the cell spreading area (CSA), from 246.8 μm^2^ for the native collagen to 216.8 μm^2^ and 163.7 μm^2^ for glycated ones, for 1 day and 5 days, respectively, and a similar trend was observed for cell perimeter 112.9 μm vs. 95.1 μm and 86.2 μm, respectively. These data suggest impaired recognition of early glycated collagen by integrin receptors. Moreover, they coincide with the reduced fibril-like reorganization of adsorbed FITC-collagen (indicating impaired remodeling) and a presumed decreased sensitivity to proteases. Indeed, confirmatory assays reveal diminished FITC-collagen degradation for glycated samples at 1 day and 5 days by attached cells (22.8 and 30.4%) and reduced proteolysis upon exogenous collagenase addition (24.5 and 40.4%) in a cell-free system, respectively. The mechanisms behind these effects remain uncertain, although differential scanning calorimetry confirms subtle structural/thermodynamic changes in glycated collagen.

## 1. Introduction

Collagen Type I is the most abundant protein in the human body and forms the main part of the extracellular matrix (ECM) within tissues. This fibrillar protein undergoes self-assembly into fibrils, a process critical for tissue mechanics and functionality [1] as fibrils act as scaffolds, supporting most cells in the body. The MSCs are undifferentiated cells [2], but they have the potential to differentiate into various cell types, typically osteoblasts, chondrocytes, adipocytes, and, in some cases, fibroblasts or myofibroblasts [3]. While residing in their niches, MSCs remain undifferentiated [4]. However, when an injury occurs, they migrate to the affected site, facilitating tissue repair [5,6]. Interestingly, MSCs are also implicated in collagen synthesis and remodeling during mechanical stretching [7]. Similar to fibroblasts, which play a major role in collagen turnover, MSCs also contribute to new collagen production and ECM organization. Morphologically, fibroblasts share mesenchymal phenotypes with stem cells but lack their differentiation potential [8]. Studies have demonstrated that MSCs can differentiate into fibroblast-like cells during tissue repair, providing an alternative mechanism for collagen production in diseases associated with impaired collagen turnover [9]. However, there remains a scarcity of parallel ex vivo studies exploring this phenomenon. In general, collagen is a thought-provoking protein with diverse functions, and its intricate interactions with the cells within the body continue to be an area of active research. The regulation of collagen synthesis and remodeling involves a sophisticated network of molecular pathways and cellular interactions, which are essential for tissue development, repair, and homeostasis. Understanding these regulatory mechanisms is critical for developing therapeutic strategies targeting collagen-related disorders and promoting tissue regeneration.

The process of collagen remodeling by MSCs is a relatively new area of research that has not been sufficiently studied in vitro. It involves several important steps: First, the continuous synthesis of new collagen to replace damaged one. The second step involves the incessant organization of this collagen, which reflects its impact on ECM formation [7]. The next step of remodeling depends on breaking old or damaged collagen by activating matrix metalloproteinases (MMPs), which are responsible for ECM degradation [10]. Presumably, MSCs are involved in regulating all these processes, including the MMPs’ activity [11]. Therefore, the true remodeling process of collagen involves the net balance between its production, organization, and degradation. Unfortunately, such complex in vitro studies are lacking, although it is becoming increasingly clear that MSCs can orchestrate collagen remodeling, ensuring tissue integrity and function. Although complexities persist, understanding these processes is essential to the advancement of regenerative medicine and tissue engineering.

Collagen synthesis occurs mostly in the cells of mesenchymal origin, typically fibroblasts, reflecting their main function. But it also occurs in MSCs, cells with the same origin [12]. It is primarily regulated by the activity of transcription factors such as Specificity Protein 1 (SP1), Activator Protein-1 (AP-1), and transforming growth factor-β (TGF-β)-induced Smad proteins, which bind to specific promoter regions of collagen genes and initiate their expression [1,9]. Additionally, post-transcriptional regulation through microRNAs (miRNAs) can modulate collagen synthesis by targeting mRNA transcripts encoding collagen proteins.

Collagen post-translational maturation occurs intracellularly and extracellularly, inextricably linked to spontaneous modifications such as glycation and oxidation. Although different types of collagens undergo different post-translational modifications, the basic outline is glycosylation [13]. Most collagens are naturally glycosylated via post-translational processing by enzymes glucosyl and galactosyl transferases, where glucosyl and galactosyl residues are covalently bonded to collagen molecules, usually to free amino groups of lysine and hydroxylysine [14]. The purpose of this glycosylation is still questionable. However, recent studies indicate that enzymatic glycosylation is important for controlling collagen secretion and possibly in the alignment of collagen fibrils, as well as in protein oligomerization [15]. The lack of activity of galactosyl transferase led to the upregulation of collagen expression and its accumulation in the endoplasmic reticulum [16]. However, subsequently, collagen can acquire covalently bound sugars, which are not supposed to be there, as a spontaneous process of non-enzymatic covalent modification [17]. This typically occurs in the tissues of diabetics, where the level of sugar is excessively high. Over time, these early glycation products (known as Amadori products) react further, forming so-called advanced glycation end products (AGEs) that are already able to make cross-links between collagen molecules [18]. This is one of the stiffening mechanisms for tissues, along with collagen non-enzymatic oxidation, aging, and additional crosslinking by overexpressed lysyl-oxidase and Rho-associated protein kinase observed in cancer [19]. AGEs are proposed as the major cause of ECM hardening associated with aging, though other ECM proteins are also involved [18]. However, cross-linking has other severe consequences, such as binding collagen molecules together, forcing them to have a fixed orientation and ultimately affecting how they are assembled into fibrils, which may provoke pathology and even cancer formation [18,20].

In the present study, we concentrate on the fate of undifferentiated MSCs in contact with ex vivo glycated collagen, and particularly on their remodeling activity. We provide morphological and quantitative (morphometric) evidence for the altered cellular interaction with glycated collagen, compiled with its significantly altered remodeling by the adhering ADMSCs, judged by their abrogated ability to reorganize adsorbed fluorescently labeled collagen. Aiming better to understand stem cells’ behavior toward glycated collagen, we also studied the cells-driven enzymatic degradation using fluorescent probes. We show its significant inhibition, a phenomenon further confirmed in a model cell-free system with exogenously added collagenase. For these studies, we chose the Adipose Tissue-Derived MSCs (ADMSCs) as a cellular model, as they combine relatively easy availability and lower donor site morbidity with characteristic multipotency, thus making them a very promising tool for tissue engineering applications [21,22].

## 2. Results

For this study, we used collagen type I derived from rat tails (RTC), which has proven its relative purity and is a useful model for various in vitro studies, including post-translational modifications. Glycation was carried out by incubation with 500 mM glucose for 1 day or 5 days (see Section 4), abbreviated below as RTC GL 1 and RTC GL 5, respectively.

### 2.1. Measurements on the Extent of Glycation

**Figure 1:**
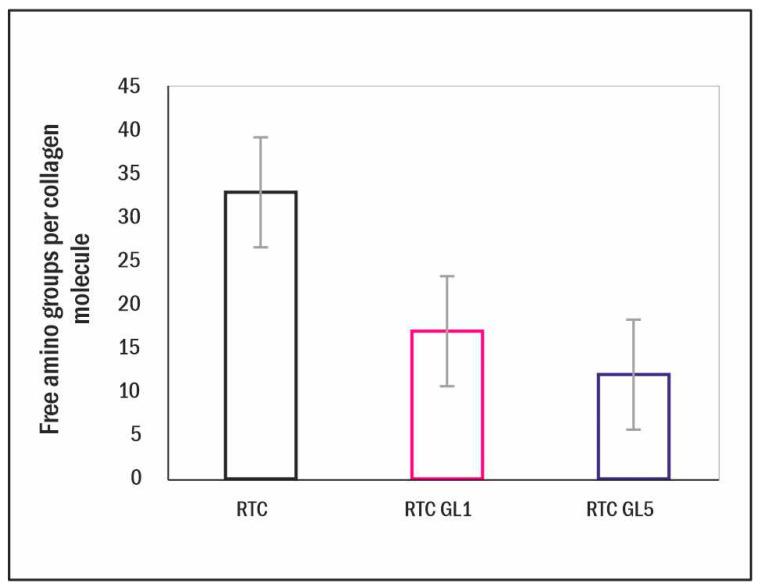
Free amino group content calculated per tropocollagen molecule for native RTC of glycated RTC for 1 day (RTC GL1) or 5 days (RTC GL5) estimated by TNBSA reaction. Quantitative determination of the number of amines contained within a sample was accomplished through comparison to a standard curve generated by using an amine of glycine dissolved in a series of concentrations from 0.1 to 0.8 mM. Bar graphs represent the standard error of the mean of the quadruplicated series.

The extent of RTC glycation was indirectly estimated using the TNBS method by measuring free and occupied lysine residues in native and glycated collagen samples. The studies of non-enzymatic glycation by mass spectrometry revealed that non-enzymatic and enzymatic glycation mainly occur in the amino acid lysine residue or its hydroxylated form [23]. This fact was used to calculate ε-amino groups of lysine residues, free and occupied by glucose. Quantitative determination of the number of amines contained within a sample was accomplished through comparison to a standard curve generated by using an amine of glycine dissolved in a series of concentrations from 0.1 to 0.8 mM. The number of free amino groups was calculated per the tropocollagen molecule. It was significantly reduced after glycation for 1 day (from 33 for the native collagen to 17 amino groups per mol), progressing to 12 amino groups per mol on the 5th day of glycation (Figure 1).

The total number of lysine residues (Lys) in an RTC molecule is about ninety; however, it should be taken into account that (i) part of the lysine residues are already hydroxylated to hydroxylysine during the initial process of enzymatic glycosylation (HyLys) necessary for fibril formation and the trafficking of collagen molecules [14]; (ii) another part of the lysine is oxidized (OxiLys) during the formation of the triple helix to develop cross-links between the collagen molecules [1,12]; therefore (iii) only the rest are actually free (Free Lys), and can participate in non-enzymatic glycation, i.e., Free Lys = [total lysine – HyLys − OxiLys]. As a result, in the native RTC, we found 33 Free Lys, approximately 1/3 of their total amount. After 1 day of glycation (RTC GL1), they drop to 17 (glycated are 33–17 = 16, e.g., approx. 48.5%), and after 5 days’ glycation (RTC GL5), they are 12 (glycated are 33–12 = 21, e.g., approx. 63.6%), presenting the actual level of non-enzymatic glycation. 

### 2.2. Differential Scanning Calorimetry (DSC) Measurements

**Figure 2:**
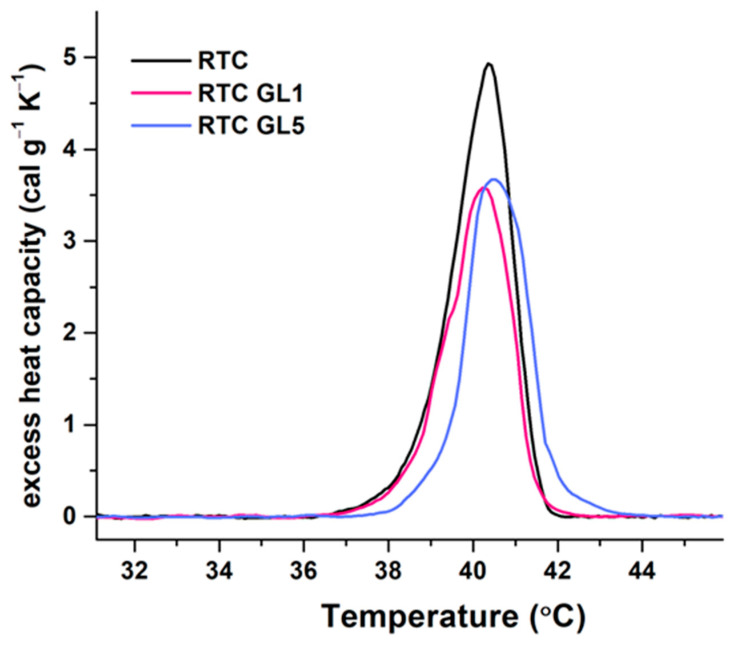
DSC profiles of native collagen (RTC, black line) and glycosylated RTC for one day (RTC GL1, red line) and 5 days (RTC GL5, blue line), respectively. The thermograms are recorded with a scan rate of 1 °C/min in the 25–55 °C range, with a 2 mg/mL collagen concentration in 0.05 M acetic acid.

Understanding the thermal stability of collagen is of great importance for its structural characterization. For this study, RTC was again glycated for 1 and 5 days as above, and after extensive dialysis versus acetic acid, the concentration was adjusted to 2 mg/mL before being analyzed for thermal denaturation upon constant heating at 1 °C/min. The representative calorimetric curves are depicted in Figure 2.

The thermal unfolding of native RTC is characterized by a single endothermic peak centered at 40.4 °C, confirming our previous study [21]. Early glycation appeared to produce weak effects on the thermal stability and slightly changed the denaturation temperature, suggesting a subtle conformational modification compared to the native RTC.

The main thermodynamic parameters (the temperature of the maximum heat capacity (Tm), the calorimetric enthalpy (ΔHcal), and the half-width of the transition (Tm^1/2^)) were further calculated from the experimental DSC curves and are presented in Table 1.

They show that after one day of glycation, the enthalpy ΔHcal decreased by 22.15% compared to native collagen (RTC native). Five-day glycation also slightly alters the thermal and conformational stability, resulting in a nearly equal decrease in enthalpy of approximately 20% relative to native RTC. Furthermore, the calculated heat capacity (ΔCp) of denaturation for both glycated samples decreased slightly by about 25–27%. It should be noted that the half-width of the transition was slightly enhanced by about 10%. Notably, the transition temperature at RTC GL5 slightly shifts to the right. However, from this result alone, it is difficult to conclude that such small thermodynamic changes can lead to a decrease in the biological activity of collagen.

### 2.3. Morphological Observations

**Figure 3:**
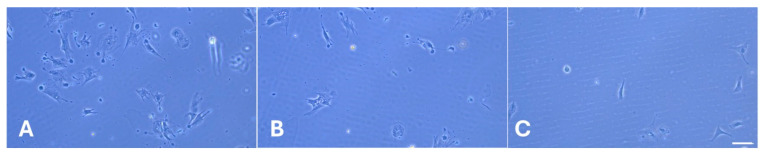
Phase contrast images of ADMSC after two hours of adhesion on native (**A**) and glycated collagens for 1 day (**B**) and 5 days (**C**), respectively. Bar 50 µm.

We coated the slides with either native collagen (RTC Nat) or that exposed to severe glycation for one day (RTC GL1) or five days (RTC GL5). Then, ADMSCs were added and cultured for 5 or 20 h. During the first 2 h, the samples were processed in a serum-free medium to avoid the effects of other adhesive factors, thus assuring that ADMSCs will attach to collagen only. Phase contrast images at that time show that both the adhesion and spreading of cells tend to be delayed on glycated collagen (Figure 3B,C), most notably on the 5-day glycated ones (Figure 3C), apart from the control RTC Nat (Figure 3A), where cells attached and spread relatively well.

**Figure 4:**
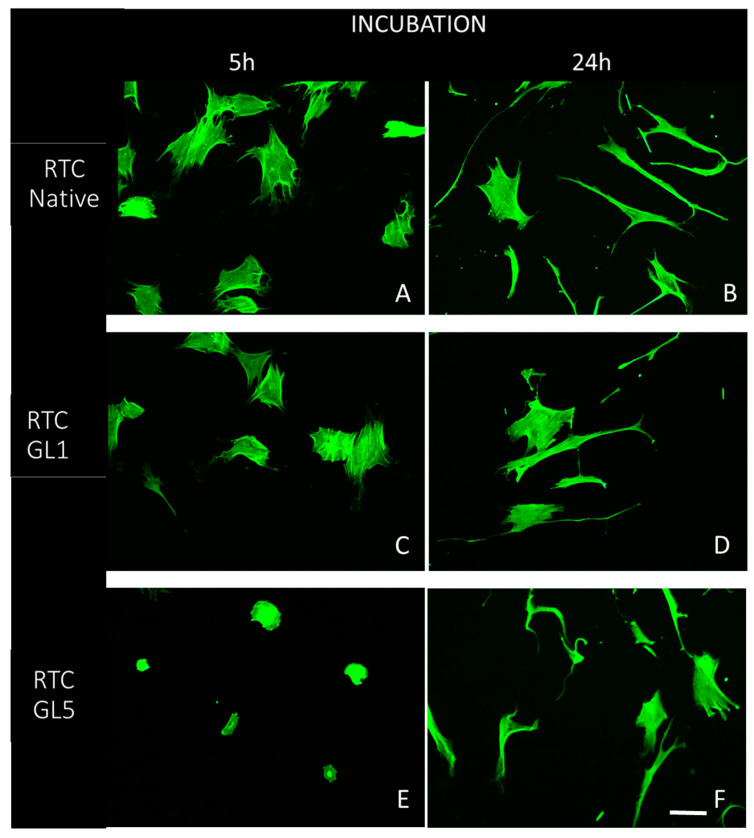
Overall cell morphology of ADMSCs adhering on native (**A**,**B**) and glycated collagens: processed for one day (**C**,**D**) or five days (**E**,**F**), respectively, viewed by the actin cytoskeleton at low magnification (20×). The left panel shows the cells at the 5th hour of incubation (**A**–**E**) while the right at the 24th hour (**B**–**F**). Bar 10 µm.

In the second hour, we added 10% serum to the medium to provide optimal ADMSC survival and functionality conditions. Parts of the samples were fixed at the 5th hour and the rest at the 24th hour before being stained for actin, vinculin, and cells’ nuclei to view the overall cell morphology, focal adhesion formations, and actin cytoskeleton development. To better characterize the overall cell shape in a comparative plan, ADMSCs were first examined at low magnification (20×), viewing the actin cytoskeleton only (green channel). Representative images are shown in Figure 4.

The study highlights that cellular interaction is more efficient on native collagen compared to glycated collagen, as evidenced by Figure 4A,B. At both the 5th and 24th hours of incubation, cells showed better spreading on native collagen, and this spread increased over time, with cells adopting an extended shape. In contrast, the spreading of ADMSCs was delayed in glycated collagen samples. This delay was less severe in the 1-day glycated samples (RTC GL1), as shown in Figure 4C,D, but became more apparent in the 5-day glycated samples (RTC GL5), as shown in Figure 4E,F. Despite the delayed spreading, cells on glycated samples eventually adopted a more extended shape over time, indicating that their functionality was partially preserved. These experiments were conducted with at least three independent series per group.

The data in Table 2 quantify the differences in cell morphology across various conditions using ImageJ 1.54g software to measure average cell spreading area (CSA), cell shape index (CSI), cell perimeter (Perimeter), and cell aspect ratio (CAR). Since the parameters were not normally distributed (as confirmed by Levene’s test), a one-way ANOVA on ranks (Kruskal–Wallis test) was used for comparisons between cells adhering to native and glycated collagen after 5 and 24 h of incubation.

Table 2 indicates that the CSA is higher in native collagen samples at both 5 and 24 h, with a significant difference observed in 5-day glycated samples at the 5th hour. Cell perimeter differences were significant at both incubation times for RTC-GL5. The CSI decreased over time, showing increased cell polarization, with significant differences at 24 h between RTC-GL1 and RTC-GL5. The CAR approximately doubled from 5 to 24 h, indicating cell elongation, with significant differences at the 5th hour between RTC and RTC-GL1.

**Figure 5:**
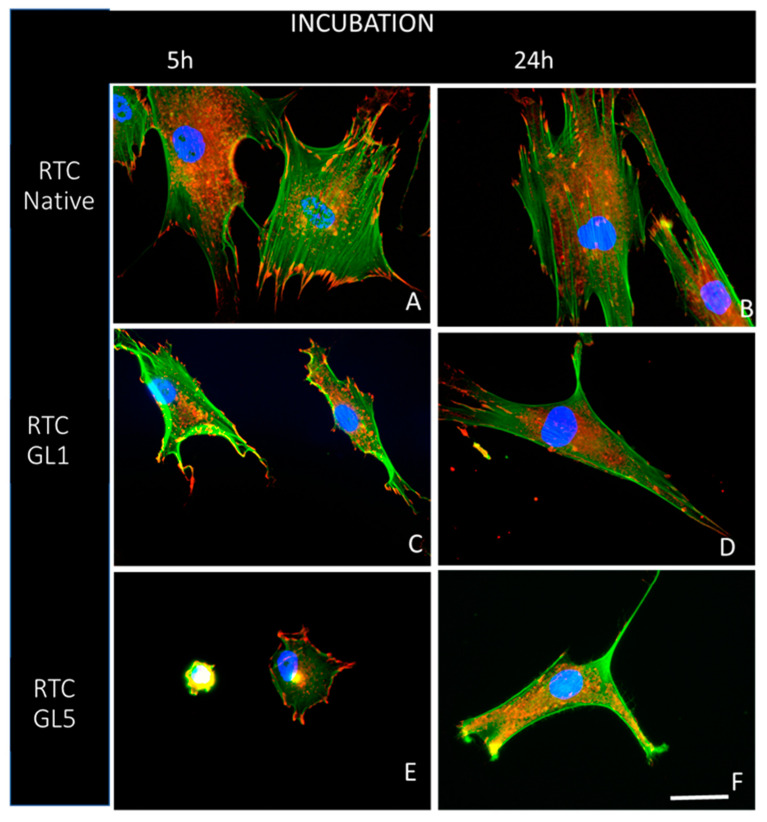
Changes in the overall morphology of cells on glycated collagen samples (representative images from a series of three experiments). The figure demonstrates via a comparative plan the development of actin cytoskeleton (green), focal adhesions (red), and the cell nuclei (blue) of ADMSC adhering on native (**A**,**B**) and glycated collagens (**C**–**F**) processed either for 1 day (designed RTC GL1 (**C**,**D**)) or for 5 days (designed RTC GL5 (**E**,**F**)), respectively. The left panel shows the overall cell morphology at the 5th hour of incubation (**A**,**C**,**E**) and the right at the 24th hour (**B**,**D**,**F**). Bar 20 µm.

These higher magnification images (Figure 5A,B) confirm again that the cells spread well on native collagen (RTC Native), valid for both the 5th and 24th hours of incubation (Figure 5A and Figure 5B, respectively), showing prominent actin stress fibers (green) inserting into the well-developed vinculin positive clusters of focal adhesions (red). This is not the case, however, for ADMSCs adhering on glycated collagens, which show significantly lower levels of focal adhesions and actin fibers development even in 1-day glycated samples (RTC GL1), at both the 5th and 24th hours of incubation (Figure 5C,D). A similar but more pronounced trend was observed for the 5-day glycated samples (RTC GL5). Interestingly, at the 5th hour of incubation, the cells showed clearly delayed spreading and almost absent actin fiber formation (Figure 5E), while at the 24th hour of incubation, the focal adhesions were less present (Figure 5E and Figure 5F, respectively). Nevertheless, even here, cell polarization progresses with the advancement of incubation time, which is valid for all samples (Figure 5B,D,F), confirming the conclusions from Figure 4 and Table 2 that ADMSCs still survive on all substrata, a fact further confirmed by the Live/Dead Assay, presented in Section 2.4 below.

To further follow the fate of adsorbed collagen, we labeled it fluorescently with FITC following the protocol of Doyle, 2021 [24], introducing some modifications, as previously described [21]. The labeled FITC-RTC was again subjected to glycation for 1 day (FITC-RTC GL1) or 5 days (FITC-RTC GL5), then coated on the substrates before being seeded with ADMSC following the same protocol as above (Figure 4).

**Figure 6:**
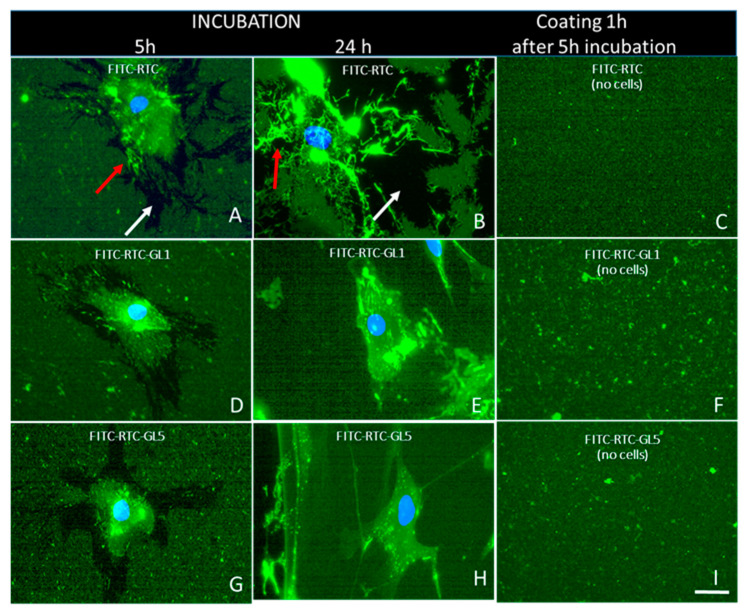
Morphological evidence for the substratum remodeling of collagen. FITC-labeled collagen (FITC-RTC) was subjected to either 1-day-glycation FITC-RTC GL1 (**D**–**F**) or 5-day glycation, FITC-RTC GL5 (**G**–**I**), and coated on glass coverslips along with controls of native FITC-RTC (**A**–**C**) before ADMSCs were added and incubated for 5 or 24 h, then fixed and stained with Hoechst to view simultaneously the adsorbed collagen (green) and the cells’ nuclei (blue), respectively, when images were merged. Images on panels (**C**,**F**,**I**) depict the plain fluorescent substrata, where no cells were added. The removed part of collagen from the substratum results in the formation of characteristic dark streaks marked with white arrows on panels (A and B). ADMSCs further rearrange collagen into a fibril-like pattern marked with orange arrows on panels (A and B). These are representative images from one out of three series of experiments. Bar 20 µm.

As can be seen in Figure 6, stem cells effectively reorganize the adsorbed native FITC-RTC, tending to remove part of it from the substratum, resulting in the formation of characteristic dark streaks, marked with white arrows in Figure 6A,B. ADMSCs further rearrange this collagen into a fibril-like pattern (orange arrows), which is already visible at the 5th hour of incubation, but progressing at the 24th hour. While no significant difference in fluorescence intensity was observed, which could be attributed to collagen glycation (as compared across images in Figure 6C,F,I), there was a certain tendency of small aggregates to form in the glycated samples (Figure 6F,I). This result generally confirms our previous observation of the stem cell-induced mechanical remodeling of collagen [21]. Glycation, however, apparently inhibits the ADMSC fibril-like remodeling, and this inhibition correlates with the extent of glycation (1 or 5 days). While few fibrils might be observed on the 1-day glycated samples (RTC GL1) at the 5th hour of incubation (Figure 6D), they were almost absent upon stronger 5-day glycation (Figure 6G), apart from the still-persisting pericellular removal of glycated collagen. A similar but more pronounced trend is seen for the 24 h cultured samples, with obviously augmented fibril-like formation, but this was clearly inhibited in glycated samples versus native ones (compare Figure 6B with Figure 6E,H), and interestingly combined with the absence of pericellular removal.

### 2.4. Cell Viability Testing

We assessed cell viability at all stages using the Live/Dead Assay, as some of the morphological findings observed in Figure 3 and Figure 4 may have been due to the altered cell vitality under these conditions. The percentage viability was calculated from images captured when cells were stained with the commercial Fluorescent Live/Dead Assay kit (see Section 4). Live cells were stained in green (Calcein positive), while dead cells were stained in red (nuclei labeled with Propidium iodide).

For reference, typical images for all conditions are provided the Data Availability Statement (Page 19) link, https://drive.google.com/drive/folders/1rxVZsvti_gc4cG6O9XR1Y2De2NLpnnUs?usp=sharing (accessed on 19 February 2024) and the corresponding calculated cell viability values (as percentages) are summarized in Table 3. At least 10 randomly chosen images were counted (in the series of two experiments), and a comparison was made with cells cultured on the regular TC polystyrene.

In Table 3, we can observe that cell viability remained relatively stable, except for a slight decline of approximately 10% at the 2nd hour of incubation. This trend aligns with the viability of ADMSCs cultured on regular tissue culture (TC) polystyrene. The initial stress resulting from enzymatic cell harvesting may account for this decrease. However, subsequent incubations with serum appear to restore cell survival.

### 2.5. Enzymatic Remodelling of Glycated Collagen

To better understand the ability of stem cells to remodel adsorbed FITC collagen, we conducted an additional experiment focusing on its enzymatic degradation by the attached cells. In this study, we utilized the phenomenon of de-quenching of fluorescent probes (known as the FRET effect), which results in the fluorescence enhancement of FITC bound to collagen upon proteolytic degradation. We employed the same approach to characterize two scenarios: first, direct cell-driven collagen degradation, and second, degradation induced by exogenously added collagenase in a “cell-free system.”

It has to be noted that this quantitative approach was successfully applied in our previous study [21,24], but here, for the samples with cells (A), we plotted the photometric signal in the presence of cells against the corresponding signal from identical controls (also with cells) but cultured on non-labeled protein (to avoid the parasitic auto-fluorescence of collagen and cells). 

**Figure 7:**
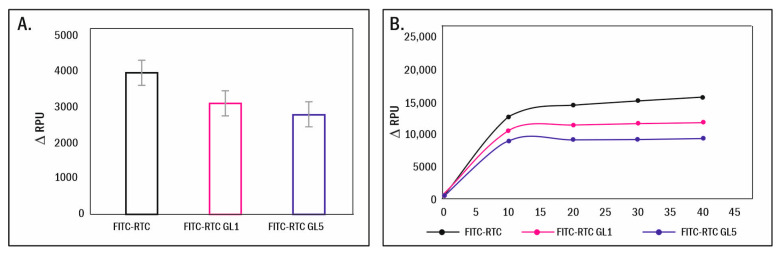
Relative changes in fluorescence of adsorbed FITC-collagen (ΔRPU) in the presence of adhering ADMSCs for 24 h (**A**) and that measured in a cell-free system (**B**) where exogenous collagenase was added. Values for native collagen (FITC-RTC) are indicated in black, while those subjected to glycation for 1 day (FITC-RTC GL1) are in red, and those glycated for 5 days (FITC-RTC GL5) are in blue. Data were obtained from three separate experiments in quadruplicated samples. Bar graphs represent the standard error of the mean. Fluorescence is given in relative photometric units (ΔRPU) (excitation 485 nm; emission 535 nm). In (**A**), ΔRPU represents the difference between the photometric signal of the “+ cells” samples versus identical controls “+ cells” but with unlabeled protein. In (**B**), the ΔRPU signal was corrected for the fluorescence of the controls obtained without collagenase.

According to the calculated ΔRPU values presented in Figure 7A, glycation significantly reduced the typical de-quenching signal from adherent ADMSCs (left column), with this inhibitory effect (22.8–30.4%) again progressing with the degree of glycation, be it 1 day or 5 days (respectively, middle and right column).

However, the cell-dependent enzymatic degradation of collagen is a process that is determined by various digestion mechanisms, including the action of soluble and membrane-bound MMPs [25]. Therefore, it was important to determine whether the above observed effects result from the altered collagen structure or altered cells’ functionality. To answer this question, we conducted the next experiment, employing a cell-free system, where exogenous collagenase was added to the collagen-coated samples. As shown in Figure 7B, exogenous clostridia collagenase expectably de-quenches native FITC-collagen (FITC-RTC), resulting in a gradual increase in the fluorescent signal until it reaches a plateau at about the 20th minute of incubation. However, this process was significantly delayed upon glycation, most pronouncedly in 5-day glycated samples (reduced by 40.4%), followed by the 1-day glycated ones (reduced by 24.52%).

## 3. Discussion

Comprehending how stem cells engage with both untreated and glycated collagen is pivotal for crafting biomaterials and engineered tissues with customized characteristics suited for particular purposes. Glycated collagen plays a central role in tissue well-being and the advancement of diseases. Hence, by delving into the nuanced interplay between stem cells and early glycation byproducts, we can unearth invaluable understandings that can inform the development of future therapies and interventions.

As stated in the introduction, glycation, also known as the Maillard reaction, occurs spontaneously when proteins react with glucose or ribose in a non-enzymatic manner [26]. Collagen, a major structural protein of the ECM that provides the mechanical integrity necessary for tissue restoration, is particularly susceptible to glycation due to its long biological half-life [27]. The interaction between glycated collagen and stem cells, whose main function is tissue repair, is crucial not only for regenerative medicine but also for understanding the pathogenesis of various diseases [28]. The biochemical process of glycation is multistage and begins with forming Schiff bases between glucose and protein amino groups [29]. Collagen was the first protein identified to undergo glycation via the ε-amino groups of lysine [30]. These unstable Schiff bases transform into Amadori products [31] and stable keto-amine intermediates, which precede the formation of advanced glycation end products (AGEs). AGEs are a subject of extensive biomedical research because of their central role in the pathogenesis of aging, the late complications of diabetes [32], vascular pathology [33,34], and cancer [35]. Despite the wealth of information on AGEs, there is a gap regarding the effects of early glycation stages—before sugar molecules react further. It is also unclear how stem cells interact with such early glycation complexes.

This study focuses on the initial interaction and remodeling of glycated collagen by MSCs upon its short ex vivo exposure to glucose (between 1 and 5 days). However, it is important to note that our glycation protocol exploits incubation with an extremely high glucose concentration (500 mM), which is approximately 100 times higher than the physiological blood concentration (about 4–6 mM); therefore, it might be considered as early, but also rather severe glycation.

To confirm that collagen is sufficiently glycated, we used the TNBS method based on decreasing the number of free lysine residues (Free Lys). Given that the total number of Free Lys is approx. 33 (out of 90 for tropocollagen molecule) at 1-day glycation (RTC GL1), they dropped to 16 (i.e., approx. 48.5%), and at 5-day glycation (RTC GL5), they were 21 (i.e., approx. 63.6%), which confirms that we have a high level of collagen glycation even at this early stage. On the other hand, it goes with an overall reduction of the free lysine residues, providing a positive electric charge to collagen molecules, which is already one mechanism that may affect cellular interaction.

Recruiting MSCs to the injury site is the first and most important step in initiating tissue repair. It includes stem cell mobilization to move from the niche to the circulation, rolling and adhesion to the vessel wall, endothelial transmigration, attachment to a distant place, etc., all of which require adhesive interactions with the ECM [6]. In this context, the adhesion to its main constituent, collagen, is of central importance; moreover, it can be easily followed ex vivo using 2D collagen-coated substrates [21]. However, this is also directly related to the interaction of stem cells with glycated collagen, which largely determines their regenerative potential. Indeed, our results truly show that the adhesion of ADMSCs to glycated collagen is substantially altered, even at this early stage of glycation, affecting primarily the initial adhesion step (2 h) with no serum in the medium (assuring cell attachment to collagen only). This raises the question: why do ADMSCs not “like” glycated collagen?

The glycation of collagen has been shown to have significant biological consequences leading to reduced cellular interaction and proteoglycan binding [33], combined with the activation of cell receptors for AGEs (RAGE), which play important roles in vascular pathology [34], diabetic complications [35] and cancer [36], as partly stated above. However, studies covering the early stages of glycation are less related to the interaction with other cell types. The pioneering work of Kawano [37] proved that early non-enzymatic glycation alters the properties of collagen as a cell adhesion substrate, causing the poor spreading of fibroblasts 3Y1. In this context, the impact of early glycation products on collagen type IV expression in mesangial cells should also be addressed [38]. More recently, it was shown that non-enzymatically glycated collagen strongly inhibited HT1080 human fibrosarcoma cells’ spreading with remarkable loss of actin stress fibers [39]. Endothelial cells cultured within glycated collagen gels also demonstrated signs of premature cell senescence, which is thought to contribute to the pathogenesis of diabetic vasculopathy [40]. However, studies involving stem cell interaction with glycated collagen are rather lacking. Moreover, little is known about the underlying mechanism. The physical stiffness of collagen fibrils may be partly responsible, though there is insufficient evidence to conclude this, as was proposed for AGEs [41]. Equally possible is that collagen glycation affects the structure of collagen via altering the accessibility to cell binding sites. However, such effects of glycation on the molecular assemblies within collagen fibrils have been studied in comparatively little detail. Collagen acts as a ligand for various substrates, including integrins, discoidin domain receptors DDR 1 and 2 [42], the leukocyte receptor complex (LRC), mannose family receptor uPARAP/Endo18, and others [43], which explains its multiple biological functions. Nevertheless, the most specific cellular interaction of type I collagen was shown to be through α2β1 and α1β1 integrin receptors [44,45], and this signal is transduced via certain adapters like Src, focal adhesion kinase (FAK), paxillin, talin, vinculin, and others, which bind to the short cytoplasmic tails of integrins [44].

Evidence suggests that integrin α2β1 and α1β1 receptors can bind to unique sequences in collagen, such as GFOGER, GXOGER, or GXOGEX (where X represents R, M, L, A, and S) [46]. The RGD sequence has also been proposed for integrin binding [47], although it was demonstrated to interact primarily with denatured collagen regions and gelatine.

However, explaining the altered adhesion of ADMSCs to glycated collagen at this recognition level poses a challenge. This is because lysine, a crucial amino acid, is not included in the above-mentioned sequences. Thus, a more plausible assumption is that glycation sterically obstructs the integrin binding sites. Notably, we revealed that a significant proportion of the free lysine molecules are glycated under our conditions (approximately 1/2 for the 1-day and 2/3 for the 5-day glycation).

Transmission electron microscopy (TEM) reveals that glycation alters both the molecular organization and charge distribution within collagen type I fibrils, particularly in the gap zone and the gap/overlap interface [48]. Consequently, the modified conformational stability of glycated collagen molecules emerges as a highly plausible mechanism.

However, the system is hardly that simple from a physiological point of view, since the recruitment of stem cells to the sites of injury is also supported by the local release of signaling molecules and growth factors [49] that promote their tropism and potentially influence the downstream expression of adhesion molecules [50]. Hence, to be more relevant, we added serum during the incubation of cells for over 2 h (for 5 and 24 h) to optimize their functionality during the anticipated remodeling of collagen, a phenomenon of primary interest [51].

While the addition of serum partially restored ADMSC morphology, the effects of glycation persisted (as observed in Figure 4). Intriguingly, under all conditions, cells exhibited robust polarization after 24 h of incubation, adopting a characteristically extended cell shape confirmed by the quantitative morphometric analysis (Table 2), which revealed nearly indistinguishable cell aspect ratios (AR) and cell shape indices (CSI).”

It has to be noted that in vivo, the polarization of stem cells plays a critical role in their recruitment to the niche, where, supported by other multiple interactions, they contribute to maintaining their quiescence [52], meaning that this ability of MSCs is rather unaffected by glycation. The effect of glycation on collagen remodeling is also remarkable (Figure 4). The typical morphological finding of FITC-collagen removal by the cells (dark zones) and its organization in a fibril-like pattern, characteristic of ADMSC when adhering to native collagen [21,23], is now strongly inhibited; moreover, it progresses with the extent of glycation. This result is difficult to compare with those in the literature, as similar studies have not been performed till recently, though a comparable inhibitory effect of collagen oxidation on its remodeling by ADMSC has been described lately by our group [21]. The removal of collagen around the periphery of cells at the 5th hour of incubation exhibited interesting characteristics with regard to glycation’s effect. It was observed that glycation did not significantly inhibit this process, except for the fibril-like reorganization. This suggests that the latter is dependent not only on motile activity but also on pericellular proteolysis, which is evidently altered during longer incubation periods. In contrast, the fibril-like organization was markedly reduced in all glycated samples, with the extent of reduction depending on both the degree of glycation and the duration of incubation (refer to Figure 6). These morphological observations, however, cannot explain the mechanism behind such impaired remodeling. Hence, a new question arises: is this due to the abrogated cellular interaction or to some other changes in the collagen molecule? Since there is a direct functional link between the mechanical and enzymatic remodeling of collagen, we decided to deepen the study by quantifying separately (1) the cell-driven enzymatic degradation and (2) the direct proteolysis caused by exogenously added collagenase in a cell-free system. For that purpose, we used the previously described approach based on the proteolytic de-quenching of fluorescent conjugated probes (FRET effect) [21]. Here, we show that the glycation significantly altered both the cell-driven (Figure 7A) and exogenous collagenase-driven (Figure 7B) enzymatic remodeling of collagen, which unequivocally indicates that the damage must be attributed to the distinct structural changes in the collagen molecule, affecting its susceptibility to proteases. The observation made here, in our view, holds potential for direct application in vivo, particularly in explaining why stem cells may fail to fully realize their regenerative capabilities, such as in the healing of wounds during advanced stages of diabetes. What is particularly noteworthy is the finding that even the products of early glycation can exert a notable inhibitory effect in this context.

However, the thermal denaturation curves (DSC) of glycated collagen (Figure 2) did not confirm this by showing new regions of transition with specific characteristics, but rather show only a light destabilization of the collagen molecule, with a small shift in the main transitional peak of about 0.2 °C. Though the detailed calculation of the total calorimetric enthalpy (∆H_cal_) and the heat capacity (ΔCp) shows a reduction of about 20–22% and 25–27%, respectively (Table 1), it is difficult to conclude that such minor structural changes alone can cause the observed decline in the biological activity. However, Reigle et al. [33] recently presented glycation zones in the tropocollagen molecule varying in composition from GKPGEQ in the α1 chain to GKPGER for the α2 chain, which may already explain why glycation disturbs the interaction with cells, as lysine (K) is in the zone of integrin binding. Hence, going back to integrins, non-enzymatic glycation presumably reduces the net positive charge of collagen molecules, thus introducing steric hindrance via glucosyl residues and consequent charge redistribution [49]. Therefore, it can be assumed that such a deviation in the intramolecular charge reduces the complementarity for interaction with integrins, with at least four (out of twenty-four) integrin heterodimers, namely, α1β1, α2β1, α10β1, and α11β1, possessing a strong affinity for collagen [44,45].

In essence, our findings indicate that the morphological changes observed in ADMSC are a result of their impaired interaction with collagen that has undergone early glycation. This impairment occurs due to steric hindrance, which prevents the proper binding of integrin receptors to complementary sequences on collagen. As a consequence, there is a decrease in the formation of focal adhesions and the development of the actin cytoskeleton, indicating compromised cell–substrate interactions that also affect collagen remodeling. Moreover, subtle alterations in the internal structure of collagen, as evidenced by changes in its thermal transition profile and significantly reduced collagen breakdown (both in a cell-driven manner and in the absence of cells after adding exogenous collagenase), further contribute to the decreased activity of stem cells in remodeling collagen, even at the early stages of glycation.

## 4. Materials and Methods

### 4.1. Collagen Procedures

#### 4.1.1. Collagen Preparation

Collagen type I was produced from the rat tail tendon by acetic acid extraction and salting out with NaCl, as described elsewhere [21]. The pellets were collected by centrifugation at 4000 rpm at 4 °C for 30 min, redissolved in 0.05 M acetic acid, and dialyzed to remove the excess NaCl. Thus, a nearly monomolecular composition of collagen solution was prepared, in which the collagen content approached 100% of the total dry mass. All procedures were performed at 4 °C. The collagen concentration in the solutions was measured by modified Lowry assay [53] and from the optical absorbance at 220–230 nm [54].

#### 4.1.2. Fluorescent Labelling of Collagen

The modified protocol of Doyle [24] was used for the FITC labeling of collagen obtained from rat tail tendons FITC-RTC. For that purpose, RTC (2 mg/mL) was dissolved in 0.05 M borate buffer (pH 8), and 20 µg of FITC (from 1 mg/mL stock in DMSO) was added per 1 mg of protein and incubated at room temperature in the dark for 90 min. Then, 0.05 M Tris buffer (pH 7.4) was used to stop the reaction, followed by extensive dialysis versus 0.05 M acetic acid, aiming to remove the excess FITC. The molar ratio FITC/Protein (F/P) was calculated from UV-VIS spectral data of FITC-RTC using the adapted formula [55] *F/P = F/C = (Amax × D)/ε*_0_ *× CM (*1*)*, where *Amax* is the absorbance of the FITC-RTC solutions measured at 494 nm; D is a dilution factor; ε_0_ is the molar extinction coefficient of FITC, equal to 70,000 M^–1^ cm^–1^; *CM* is the molar collagen concentration.

#### 4.1.3. Preparation of Glycated Collagen

Collagen type I obtained from rat tail tendon—RTC (2 mg/mL)—was pre-glycated by incubation with 500 mM of glucose solution (Merck, Rahway, NJ, USA) in PBS at pH 7.4, containing 0.02% NaN_3_ for 1 and 5 days at 37 °C as described elsewhere [56]. The samples were dialyzed versus 0.05 M acetic acid before being designed as RTC GL1 and RTC GL5, respectively, and stored at 4 °C until use.

### 4.2. TNBS Method for Quantifying Free Amino Groups

2,4,6, Trinitrobenzene sulfonic acid (TNBS) interaction was used to quantify the number of free amino groups in native and glycated collagen samples, according to Fields’ original method [57]. In short, collagen samples (0.5 mL) were dissolved in 0.1 M sodium bicarbonate, pH 8.5 at 100 μg/mL concentration. Freshly prepared 0.01% (*w*/*v*) TNBSA (0.25 mL) (Thermo Fisher Scientific, Waltham, MA, USA) was added, and samples were incubated at 37 °C for 2 h. The reaction was visualized by adding 0.25 mL of 10% SDS and 0.125 mL of 1 N HCl to each sample. The absorbance of the solutions was measured at 335 nm. The quantitative determination of the number of amines within samples was estimated by comparison to a standard curve generated by glycine (2–20 μg/mL). Each value is the mean of the quadruplicated experiment.

### 4.3. Cells

Human adipose tissue-derived mesenchymal stem cells (ADMSC) of passage 2 were received from Tissue Bank BulGen (Sofia, Bulgaria), prepared after obtaining regular volunteers’ written consent before liposuction. The cells were maintained in DMEM/F12 medium containing 1% GlutaMAX™, 1% Antibiotic-Antimycotic solution, and 10% fetal bovine serum (FBS), all purchased from Sigma Aldrich (St. Louis, MO, USA). Every two days, the medium was replaced until the cells reached approximately 90% confluency to be used for the experiments up to the seventh passage.

For each experiment, the viability of ADMSCs was verified by trypan blue exclusion testing upon harvesting the cells. Over 85% vitality was accepted as permissible for each experiment. For more specific studies of attached cells, the Live/Dead Assay Kit was used (see Section 4.3.5).

#### 4.3.1. Morphological Studies

For the morphological observations, collagen (100 μg/mL) dissolved in 0.05 M acetic acid was used to coat regular glass coverslips (12 × 12 mm, ISOLAB Laborgeräte GmbH, Eschau, Germany), which were incubated for 60 min at 37 °C and placed in 6-well TC plates (Sensoplate, Greiner Bioone, Meckenheim, Germany). After 3 washes with PBS, the cells were seeded at density 5 × 10^4^ cells/well in the final volume of 3 mL serum-free medium before being incubated for 5 and 24 hours. At the 2nd hour, 10% FBS was added. The initial cell adhesion and morphology were studied at the 2nd hour under phase contrast at magnification 20X using an inverted microscope, Leica DM 2900, and further incubated up to the 5th or 24th hour before being processed for immunofluorescent staining and morphometric analysis, as follows.

#### 4.3.2. Overall Cells Morphology and Focal Adhesion Formation

After 5 or 20 h of incubation, the samples were fixed with 4% paraformaldehyde and permeabilized with 0.5% Triton X-100 before fluorescence staining. Green fluorescent Alexa fluorTM 488 Phalloidin (Invitrogen, Thermo Fisher Scientific Inc Branchburg, NJ, USA) was used to visualize the actin cytoskeleton, while the cell nuclei were stained with Hoechst 33342 (dilution 1:2000) (Sigma-Aldrich/Merck KGaA Darmstadt, Germany). Focal adhesions were viewed with Anti-Vinculin Mouse Monoclonal Antibody (Clone: hVIN-1, Thermo Fisher Scientific, Waltham, MA, USA) IgG in dilution 1:150, followed by fluorescent Alexa Fluor 555 conjugated goat anti-mouse IgG antibody (both provided by Sigma-Aldrich) used in dilution 1:100.

#### 4.3.3. Quantitative Morphometry Analysis of Raw Format Images by ImageJ

All image analyses were performed per cell using ImageJ 1.54g software version, which provides a wide range of processing and analysis approaches [58]. The fluorescence intensity was measured based on raw format images of cells at magnification 20X captured from at least three separate images under the same conditions. Pixel-based treatments were performed to highlight the regions of interest (ROIs) and allow the removal of artifacts. A default black-and-white threshold was used in the segmentation module. Images of equal sizes (W: 1600 px/H: 200 px) were examined, and four metrics were acquired, namely, spread area (SA), cell shape index (SCI), and aspect ratio (AR). For that purpose, the individual cellular domains were determined by generating binary masks using Otsu’s intensity-based thresholding method from 20× fluorescent actin images. Cellular masks were then used to calculate ADMSC SA, CSI, and AR. The CSI was calculated using the formula

CSI = 4π × A/P^2^

where A is the mean cell area, and P is the mean cell perimeter.

With this metric, a line takes a CSI value of zero (indicating an elongated polygon) or one (indicating a circle). AR was calculated as the ratio of the largest and smallest sides of a bounding rectangle encompassing the cell. These morphometric data relating to all experimental conditions, including the number of cells examined, are summarized in Table 2.

#### 4.3.4. Measurement of Cell Survival

For that purpose, the Live/Dead Assay Kit 04511 from Sigma-Aldrich was used, providing fluorescence double staining of live and dead cells based on the simultaneous treatment of adhering non-fixed cells with Calcein AM and propidium iodide. Following the manufacturer’s protocol, the cytosol of living cells was stained green, and the nucleus of dead cells was stained red. Representative images were captured on an inverted fluorescent microscope, Leica DM 2900, as above, using 20× magnification, and the % of the dead cells was estimated from at least ten (10) images.

#### 4.3.5. Measurement of Collagen Degradation by ADMSC

The collagen degradation assay relies on the de-quenching phenomenon of fluorescently labeled protein, commonly known as the FRET effect [59].

Initially, collagen is labeled with FITC in excess, which results in the partial shadowing or quenching of the FITC signal due to the high density of FITC molecules on the collagen surface. When the protein undergoes degradation (e.g., enzymatic breakdown), the FITC molecules within the collagen structure move apart, and the quenching effect diminishes, leading to a distinct increase in fluorescence intensity.

In summary, the FRET effect provides valuable insights into collagen dynamics and proteolytic activity, making it a powerful tool for studying cellular processes.

Briefly, 24-well glass-bottomed black SensoPlates TM (Lab Logistics Group GmbH, Meckenheim, Germany) were pre-coated with native FITC-RTC (control), or 1-day glycated (FITC-RTC GL1) or 5-day glycated (FITC-RTC GL5) collagen solutions (0.100 μg/mL), then washed three times with PBS before cells were added (1 × 10^4^ per well) into a final volume of 1 mL serum-free medium (conditions assuring single protein adhesion of cells to collagen). After 2 h of incubation, 10% serum was added, and the cells were further cultured for 5 or 20 h in a humidified CO_2_ incubator. The adsorbed collagen layer was measured directly from the bottom of the plate (in 1 mL PBS) using a Multimode Microplate Reader (Mithras LB 943, Berthold Technologies GmbH & Co. KG, Bad Wildbad, Germany) set at 485/535 nm. Control samples also with adhering cells but coated with non-labeled collagen (RTC, RTC GL1, or RTC GL5) were processed in the same way. All experiments were quadruplicated. Fluorescence intensity is presented as relative photometric units (RPUs) subtracted from the signal from non-labeled control samples.

### 4.4. FITC-Collagen Degradation in Cell-Free System

Quadruplicated samples of FITC-RTC, FITC-RTC G L1, and FITC-RTC GL5 coated substrata were prepared in the same 24-well glass-bottomed TC plates using the standard protocol (incubation for one hour with 100 µg/mL collagen solution in 0.05 M acetic acid followed by 3 PBS washes). Then collagenase type I from *Clostridium histolyticum* (Genaxxon Bioscience GmbH. Ulm., Baden-Württemberg, Germany) at 3.7 mg/mL in TC medium was added to the samples before being incubated for 60 and 120 min at 37 °C. The fluorescence of the quadruplicated samples was measured with a Multimode Microplate Reader (as above) set to excitation/emission wavelengths of 485/535 nm. Fluorescence intensity obtained as relative photometric units (RPUs) was presented as ∆RPU (reflecting the difference in the fluorescent signal between samples with collagenase vs. controls without collagenase).

### 4.5. DSC Measurements

DSC measurements were performed using a DASM4 (Privalov, BioPribor, Moscow, Russia) built-in, high-sensitivity calorimeter with a cell volume of 0.47 mL. The collagen concentration was adjusted to 2 mg/mL in 0.05 M acetic acid. A constant pressure of 2 atm was applied to the cells to prevent any degassing of the solution. The samples were heated with a scanning rate of 1.0 °C/min from 20 °C to 65 °C and preceded by a baseline run with buffer-filled cells. Each collagen solution was reheated after cooling from the first scan to evaluate the reversibility of the thermally induced transitions. The calorimetric curve corresponding to the second (reheating) scan was used as an instrumental baseline and was subtracted from the first scans, as collagen thermal denaturation is irreversible. The calorimetric data were analyzed using the Origin Pro 2018 software package.

### 4.6. Statistical Analysis 

All experiments were conducted in at least three independent series per group. Since the morphometry parameters were not normally distributed (as confirmed by Levene’s test), a one-way ANOVA on ranks (Kruskal–Wallis test) was used as it is more appropriate for comparisons between subgroups of cells adhering to native and glycated collagen.

Subgroup differences in RTC, RTC-GL1, and RTC-GL5 samples were studied using Dunn’s test. The Standard Error of the Mean (SEM) of the colorimetric TNBS measurements data is presented in bar graphs, and the same is given for the data of fluorescent FITC collagen degradation. Significance is indicated by asterisks (*), corresponding to *p* < 0.05.

## Figures and Tables

**Table 1:** Thermodynamic parameters: transition temperature (T_m_), total calorimetric enthalpy (∆H_cal_), and transition half-widths (T_m_ ½) obtained from the DSC profiles of the native RTC and RTC glycated for 1 day (RTC GL1) and 5 days (RTC GL5).

Sample	T_m_ (°C)	∆H_cal_ (cal g^−1^)	c_P_^ex^ (cal.g^−1^ K^−1^)	T_m_ ½ (°C)
RTC native	40.4	8.76	4.93	1.57
RTC GL1 (1 day glycated)	40.2	6.82	3.58	1.72
RTC GL5 (5 days glycated)	40.5	6.98	3.67	1.71

**Table 2:** Morphometric parameters characterizing the spreading of ADMSCs on native and glycated collagens. The table presents the morphometric parameters used to quantify the spreading of ADMSC adhering to native and glycated collagens. The parameters include: (i) cell spreading area (CSA) in μm^2^; (ii) mean cell perimeter (Perimeter) in μm; cell shape index (CSI) and cell aspect ratio (CAR). Values are given as averages ± Standard Error of the Mean (SEM), with the number of cells studied indicated in parenthesis. The Kruskal–Wallis test was used to determine significant differences between the subgroups of native collagen (RTC) and glycated collagen samples, glycated for 1 day (RTC-GL1) and 5 days (RTC-GL5) within 5 h and 24 h incubation groups. Dunn’s test was employed to identify significant differences for each parameter within the subgroups. Statistically significant differences (*p* < 0.05) are marked with asterisks (*), and small letters (a, b, c, d, e) indicate which values were compared.

Parameter	Incubation Time	RTC	RTC-GL1	RTC-GL5
CSA (μm^2^)	5 h	246 ± 143 (*n* = 45) *a	217 ± 146 (*n* = 42)	164 ± 109 (*n* = 40) *a
	24 h	249 ± 150 (*n* = 34)	211 ± 171 (*n* = 32)	226 ± 123 (*n* = 20)
Perimeter (μm)	5 h	113 ± 56 (*n* = 30) *b	95 ± 40 (*n* = 42)	86 ± 42 (*n* = 40) *b
	24 h	130 ± 54 (*n* = 34) *c	72 ± 5 (*n* = 30)	65 ± 4 (*n* = 30) *c
CSI	5 h	0.30 ± 0.21 (*n* = 45)	0.3 ± 0.2 (*n* = 45)	0.3 ± 0.1 (*n* = 20)
	24 h	0.21 ± 0.12 (*n* = 34)	0.23 ± 0.17 (*n* = 34) *d	0.14 ± 0.11 (*n* = 20) *d
CAR	5 h	1.5 ± 0.4 (*n* = 45) *e	1.9 ± 0.9 (*n* = 42) *e	1.7 ± 0.6 (*n* = 40)
	24 h	3.4 ± 0.23 (*n* = 34)	3.6 ± 2.5 (*n* = 32)	3.9 ± 2.7 (*n* = 20)

**Table 3:** Cell viability was tested for all stages of the experiment using Live/Dead Assay (typical images are presented in Data Availability Statement). Comparison at all points was done with cells cultured on regular TC polystyrene. The percentage of viability and the total number of counted cells are shown in brackets for all conditions.

Incubation Time in Hours	2 h	5 h	24 h
Viability (%) (Number of viable cells)	Live Cells	Live Cells	Live Cells
RTC native	94.2 (114)	95.4 (165)	96.2 (230)
RTC GL1	88.4 (122)	94.8 (251)	95.1 (330)
RTC GL5	74.6 (94)	94.7 (270)	93.3 (252)
TC Polystyrene	80.5 (124)	88.5 (193)	94.5 (329)

## Data Availability

The morphology data used for the morphometric analysis are available via the link https://drive.google.com/drive/folders/1rxVZsvti_gc4cG6O9XR1Y2De2NLpnnUs?usp=sharing (accessed on 19 February 2024), other data available on request from the corresponding author.

## Abbreviations

Adipose-derived mesenchymal stem cells (ADMSC), Matrix Metalloproteinases (MMPs), Rat Tail Tendon Collagen (RTC), 1-day-glycated RTC sample (RTC GL1), 5-day-glycated RTC sample (RTC GL5), Specificity Protein 1 (SP1), Activator Protein-1 (AP-1), Transforming growth factor-β (TGF-β), Advanced Glycation End-products (AGEs), Trinitrobenzene sulfonic acid (TNBS). RGD, GKPGEQ, GKPGER, GFOGER, GXOGER, and GXOGEX, L, S are amino acid sequences abbreviated as follows: (A—alanine, D—aspartate, G—glycine, E—glutamate, R—arginine, K—lysine, L—leucine, P—proline, O—hydroxyproline, Q—glutamine, S—serine).
